# Case Report: Massive subcapsular hepatic hematoma after spontaneous rupture of pyogenic liver abscess in a healthy young woman

**DOI:** 10.3389/fmed.2025.1713210

**Published:** 2026-01-08

**Authors:** Jiaqi Chen, Zejin Zhao, Kunyu Huang, Liyong Zhang, Jinhua Cui, Ziyu Bai, Aijun Yu, Jian Li, Zhuqing Zhang, Kai Chen

**Affiliations:** 1 Department of Hepatobiliary Surgery, The Affiliated Hospital of Chengde Medical University, Chengde, Hebei, China; 2 Hebei Key Laboratory of Panvascular Diseases, Department of Hepatobiliary Surgery, Affiliated Hospital of Chengde Medical University, Chengde, Hebei, China; 3 Department of Clinical Laboratory Services, The Affiliated Hospital of Chengde Medical University, Chengde, Hebei, China

**Keywords:** case report, laparoscopic surgery, rupture of hepatic abscess, septic shock, subcapsular hepatic hematoma

## Abstract

Bacterial and parasitic agents can cause liver abscess (LA), and spontaneous rupture of a pyogenic liver abscess (PLA) represents an exceedingly rare but life-threatening event. Upon abscess rupture, a sudden influx of pus and bacterial toxins into the peritoneal cavity can trigger a dysregulated host response to infection. Should major intrahepatic vessels be involved, life-threatening hemorrhagic shock may supervene, with clinical deterioration occurring within hours. We present the first case of a young woman with a ruptured liver abscess complicated by a massive subcapsular hematoma who rapidly developed hemorrhagic and septic shock and underwent emergency laparoscopic surgery followed by intensive care unit (ICU) admission. No adverse complications were observed in follow-ups at one, three, and six months after the operation. This case emphasizes that even immunocompetent young adults can experience acute abscess rupture with life-threatening hemorrhagic shock, mandating early recognition, prompt intervention, and multidisciplinary management.

## Highlights

- We present the first case of a young woman with a ruptured liver abscess complicated by a massive subcapsular hematoma who rapidly developed hemorrhagic and septic shock and underwent emergency laparoscopic surgery.
- Contrast-enhanced computed tomography (CECT) imaging at the 6-month post-operative follow-up confirmed complete resolution of the abscess without residual inflammation or complications, underscoring the durability of the laparoscopic intervention.
- Our case provides a referable therapeutic strategy for ruptured liver abscess and delineates the changes in immune-inflammatory indices during abscess rupture.
- This case emphasizes that even immunocompetent young adults can experience acute abscess rupture with life-threatening hemorrhagic and septic shock, mandating early recognition, prompt intervention, and multidisciplinary management.
- By comparing the dynamic changes in infection markers before and after treatment for ruptured liver abscess, this case extends to explore the mechanisms and translational value of immunology and microbiology in abdominal septic shock.

## 1 Introduction

LA constitutes a major clinical challenge, characterized by a localized infection within the hepatic parenchyma that can result in substantial morbidity and mortality. Common complications include pneumonia, pleural effusion, sepsis (4), septic shock, and multiple organ dysfunction syndrome (5). However, rupture of a liver abscess with subsequent bleeding resulting in a large subcapsular hematoma is exceedingly rare. A systematic review of the relevant literature (6, 7) identified several cases of ruptured liver abscess. Nevertheless, none described the formation of a subcapsular hematoma, and all were managed solely by conventional open surgery or percutaneous transhepatic drainage alone. We first report an exceedingly rare case of a ruptured and hemorrhagic pyogenic liver abscess in a previously healthy young woman who underwent emergent laparoscopic débridement and hemostasis with excellent results, aiming to offer a referable therapeutic strategy for the management of ruptured liver abscess and to emphasize translational insights into the immune response during intra-abdominal abscess rupture. The details are presented below.

## 2 Case history

### 2.1 General information

**Figure 1:**
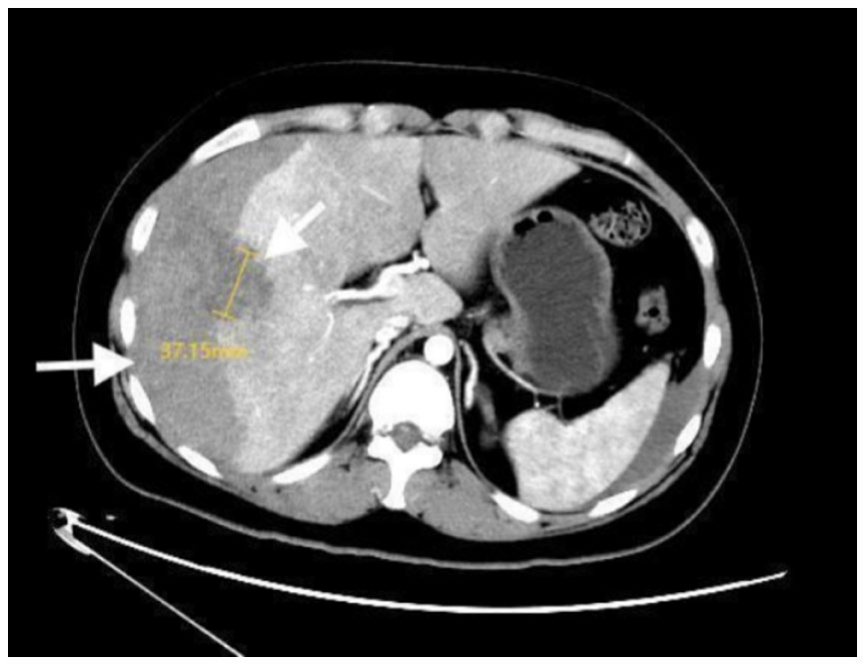
Contrast-enhanced abdominal CT images obtained on admission. Contrast-enhanced abdominal CT shows a 37.15 mm right-lobe abscess (left arrow) with massive subcapsular hematoma (right arrow).

A 24-year-old woman was admitted to the hospital due to “fever and right upper quadrant pain” for two days. She was unmarried and nulliparous, and denied any history of hepatobiliary disease, metabolic disorders, immunocompromise, or alcohol or drug abuse. Vital signs were as follows: BP 111/70 mmHg, RR 31 breaths per minute, PR 130 beats per minute, and temperature 36.0 °C. Additionally, physical examination revealed a pale complexion, cold and clammy extremities, and positive abdominal tenderness with mild rebound pain. Emergency contrast-enhanced abdominal computed tomography (CECT of the abdomen) showed: probable subcapsular hepatic hematoma, possible liver abscess, hepatic injury, and suspected hemoperitoneum (Figure 1). Initial laboratory tests revealed: white blood cell (WBC) 22.87 × 10^9^/L with neutrophils (NEUT) 89.4%, C-reactive protein (CRP) 230.32 mg/L, procalcitonin 6.492 ng/mL; hemoglobin (HGB) 106 g/L; total bilirubin level (TBIL) 106.57 μmol/L (normal range 3–22); hepatocellular enzyme elevation [alanine aminotransferase (ALT) 177 U/L (normal range 0–35) and aspartate aminotransferase (AST) 264 U/L (normal range 14–36)]. A provisional diagnosis of liver abscess with hepatic hemorrhage and hemoperitoneum was made. We supposed that intraperitoneal bleeding was secondary to the rupture of an abscess. While rapidly administering intravenous fluids and broad-spectrum antibiotics for septic shock, we proceeded to emergency laparoscopic débridement and hemostasis of the hepatic tear, drainage of the liver abscess, and thorough peritoneal lavage with drain placement.

### 2.2 Surgical procedures and process

**Figure 2:**
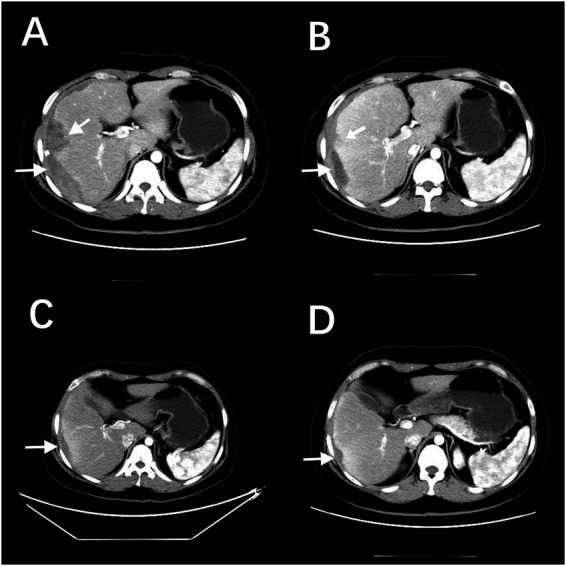
Intra-operative laparoscopic view. **(A)** Laparoscopic view demonstrating a massive subcapsular hematoma of the right liver (arrow). **(B)** After evacuating part of the hematoma, a 4 × 4 cm hepatic abscess cavity was exposed (arrow). **(C)** Continue to dissect and evacuate the subcapsular hematoma of the liver. **(D)** The processed hepatic raw surface.

General anesthesia with endotracheal intubation was instituted, and preoperative antibiotics were administered. Laparoscopy view revealed a massive subcapsular hematoma of the right liver measuring approximately 15 × 16 cm (Figure 2A), with an actively bleeding capsular tear along the inferior hepatic border, hemoperitoneum with blood and clots accumulating in the perihepatic, perisplenic, and pelvic spaces, as well as omental adhesion to the site of hepatic rupture. After adhesiolysis with a disposable ultrasonic scalpel and partial evacuation of the subcapsular hepatic hematoma using an aspirator, a 4 × 4 cm hepatic abscess cavity was exposed in the right lobe (Figure 2B), exhibiting active bleeding, internal septations, and purulent fluid. Approximately 2,500 mL of blood and clots were aspirated from the perihepatic, perisplenic, and pelvic areas.

Further meticulous dissection and evacuation of the hematoma were performed (Figure 2C). Hemostasis of the liver surface was then achieved using a disposable bipolar device and an electrocautery hook, with titanium clips applied to bleeding vessels for additional hemostatic control (Figure 2D). Subsequently, the evacuated hematoma was placed into a specimen retrieval bag and removed intact through the right upper abdominal trocar site. The hepatic injury surface and the abdominopelvic cavity were thoroughly irrigated with a large volume of normal saline. After confirming the absence of active bleeding or bile leakage, negative-pressure drainage tubes were placed in the subhepatic space and anterior to the hepatic abscess cavity, and exteriorized through the abdominal wall. At this stage, estimated intra-operative blood loss was 500 mL, and a concomitant hemoglobin level of 86 g/L confirmed ongoing hemorrhage. Therefore, the patient received 4 units (800 mL) of leucocyte-depleted B-positive red blood cell concentrate plus 400 mL of fresh-frozen plasma during the procedure.

### 2.3 Results

**Figure 3:**
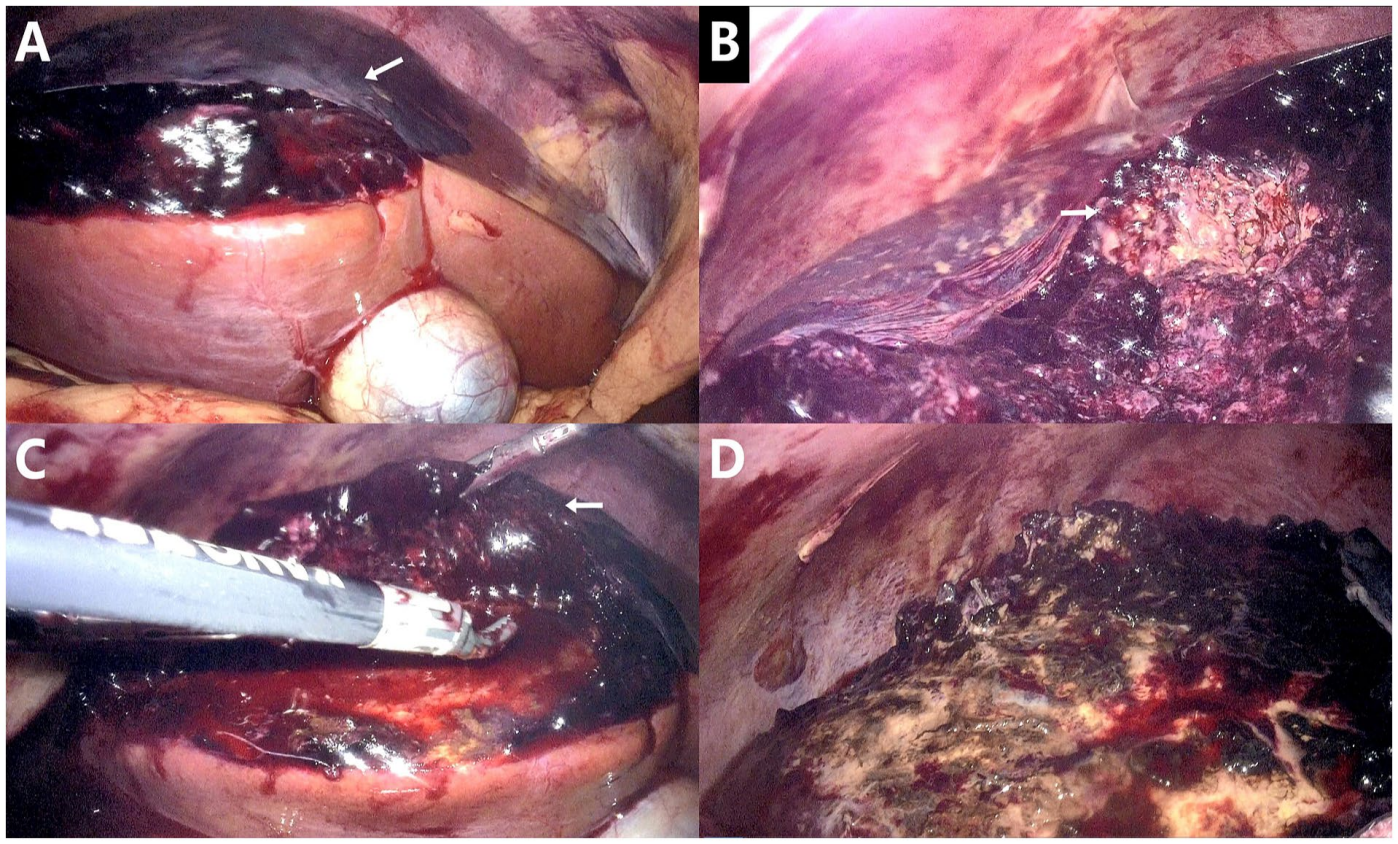
Post-operative follow-up contrast-enhanced CT (CECT). **(A)** One-week postoperative CECT. **(B)** One-month postoperative CECT. **(C)** Three-month postoperative CECT. **(D)** Six-month postoperative CECT. Follow-up imaging documents stepwise shrinkage and ultimately complete resolution of the abscess.

Given the patient’s critical condition on admission, she was transferred to the ICU immediately after surgery. The APACHE II score was 15 (8), and she received respiratory support, blood transfusion, albumin supplementation, and other comprehensive treatments. On the first day after ICU admission, the patient developed progressively worsening dyspnea (RR 35 breaths/min). Chest X-ray revealed bilateral pulmonary infiltrates, and arterial blood gas analysis showed FiO₂ 41%, PaO₂ 47 mmHg, PaCO₂ 36 mmHg, PaO₂/FiO₂ <150, consistent with the Berlin criteria for acute respiratory distress syndrome (ARDS) and type I respiratory failure (9). Hence, high-flow oxygen therapy (FiO₂ 65%, flow rate 50 L/min), antimicrobial treatment, and albumin combined with diuretics were initiated promptly. Repeat chest X-ray demonstrated improvement in the bilateral pulmonary infiltrates 2 days later, with SpO₂ increasing to 95%. She also reported a marked alleviation of dyspnea. Based on the postoperative chest CT findings—including pulmonary inflammation, bilateral pleural effusions, and areas of atelectasis—we considered the ARDS to be primarily attributable to pulmonary infection. The pleural effusions were likely related to hypoproteinemia secondary to hemorrhage, while the pneumonia may have been secondary to the hepatic abscess or associated with ventilatory support. After 4 days of comprehensive supportive therapy, the patient showed sufficient clinical improvement to be transferred back to our department. On the following day, we performed a dynamic liver function test (DDG-530X); the 15-minute indocyanine-green retention rate (ICG-R15) was 2.6% (normal range <10%), indicating adequate hepatic functional reserve. Besides, contrast-enhanced computed tomography (CECT) at 1 week post-operation also revealed a marked reduction in the lesion (Figure 3A). Meanwhile, symptomatic treatment with ulinastatin, cefoperazone-sulbactam sodium, and magnesium isoglycyrrhizinate injection was administered, and liver function, inflammatory indices, and routine blood tests had essentially normalized by postoperative day 12 (HGB 112 g/L, TBIL 25.7 μmol/L, procalcitonin 0.102 ng/mL, CRP 37.41 mg/L), allowing the patient to be discharged home. Post-discharge surveillance CECT at 1, 3, and 6 months showed progressive involution and complete resolution of the abscess (Figures 3B–D), with no adverse complications observed.

## 3 Discussion

PLA, a suppurative infection of the hepatic parenchyma, is the predominant subset among hepatic infectious lesions of diverse etiologies (including amoebic, fungal, echinococcal, etc.) (1, 7). Its escalating incidence has made PLA a leading cause of hospitalization and mortality in low- and middle-income countries, constituting an increasingly serious public-health challenge (10, 11). Among these complications, abscess rupture represents a rare yet frequently fatal event (3). Nevertheless, published cases of ruptured liver abscess have predominantly involved patients with recognized risk factors such as diabetes mellitus, underlying hepatobiliary pathology, gastrointestinal malignancy, or advanced age (12). The present case is the first to document that even young, previously healthy individuals without comorbidities can present with the catastrophic constellation of “rupture-hemorrhage-septic shock” due to PLA, underscoring the imperative for early recognition and prompt multidisciplinary intervention.

PLA is a potentially life-threatening condition, with published mortality rates reaching 31%. Historically, hematogenous spread from distant foci was regarded as the principal route of hepatic seeding (13). Recent evidence, however, indicates that biliary tract lesions—such as cholelithiasis, strictures, neoplasms, or congenital anomalies—have supplanted hematogenous dissemination as the leading etiology, making biliary tract infection the most commonly implicated pathogenic mechanism (7). According to the relevant literature, the predominance of right lobe involvement (75.9%) observed aligns with anatomical studies exploring the preferential blood flow to the right lobe (14). Although biliary tract infection is still regarded as the commonest causative mechanism, pathogens may also reach the liver via the hepatic artery or portal vein from gastrointestinal or other remote foci, or may superinfect pre-existing hepatic lesions. Furthermore, a considerable proportion of cases remain cryptogenic in clinical practice (13). At present, the routes by which infection reaches the liver are categorized into five principal mechanisms: hematogenous dissemination through the hepatic artery, portal vein, or umbilical vein, as observed in metastatic septic abscess, portal-vein thrombosis, or omphalitis; biliary spread secondary to cholecystitis, cholangitis, as well as from invasion by parasites or foreign bodies; the spread of inflammatory processes to the liver from surrounding areas; post-traumatic infection following hepatic injury or intrahepatic hematoma; and post-operative infection (7). Given that the patient was young, we initially considered the possibility that the subcapsular hepatic hematoma might have resulted from blunt abdominal trauma with secondary necrosis or infection. However, detailed history-taking and thorough physical examination revealed no skin bruising, abdominal wall contusions, or any other signs suggestive of trauma, thereby largely ruling out traumatic injury as the underlying cause. Although a subcapsular hepatic hematoma was indeed observed intraoperatively, its morphology and location were more consistent with a secondary change resulting from abscess rupture rather than trauma-induced injury. On the other hand, it should be noted that the exclusion of trauma relied partly on the patient’s self-reported history, which may introduce an inherent limitation.

Rupture of a liver abscess is a rare yet frequently fatal complication. In the study by Chou et al. (6) of 424 patients with pyogenic liver abscess, the incidence of abscess rupture was only 5.4%, but the associated mortality rate reached 43.5%. Particularly when diagnosis is delayed, and treatment is neglected, mortality rates as high as 75% have been reported (15). Consequently, early recognition and prompt institution of appropriate therapy are critical. Nevertheless, pyogenic liver abscess often has an insidious onset, presenting initially with non-specific fever and chills, followed by right upper quadrant pain and hepatic tenderness (16). Laboratory findings are equally non-specific; although leukocytosis and elevated alkaline phosphatase are common, they contribute little to establishing the diagnosis (17). In the absence of imaging confirmation (ultrasound or CT), the condition is easily missed. Therefore, early confirmation of the lesion by imaging is pivotal to prevent complications such as abscess rupture and a dysregulated host response to infection (2, 18, 19). Therefore, early confirmation of the lesion by imaging is pivotal to prevent complications such as abscess rupture and a dysregulated host response to infection.

Pyogenic liver abscess is typically linked to biliary infection and systemic sepsis, exhibiting a polymicrobial etiology with frequent isolation of organisms such as *Klebsiella pneumoniae* and *Escherichia coli* (20, 21). Although spontaneous rupture of liver abscess is uncommon, the incidence is significantly higher in abscesses caused by *Klebsiella pneumoniae* compared with those of other etiologies. Among patients with *Klebsiella pneumoniae* liver abscess, persistent hyperglycemic dysregulation in diabetes mellitus, abscess diameter greater than 5 cm, a thin abscess wall, and gas-forming abscesses—often accompanied by right upper quadrant pain and hepatic tenderness—are recognized risk factors for spontaneous rupture (22). Diabetic individuals are highly susceptible to liver abscess because chronic hyperglycemia impairs neutrophil chemotaxis and phagocytic function (13), thereby compromising host immunity and increasing the risk of infection (23). Microbiological etiological diagnosis largely depends on blood cultures and pus cultures. However, discrepancies may occur between blood and abscess cultures, which can be attributed to multiple contributing factors, including early antibiotic administration, differences in bacterial load and growth conditions, and variations in specimen-collection technique. Studies have shown that early antibiotic exposure before drainage reduces the bacterial recovery rate from abscess cultures, and technically challenging hepatic abscess aspiration with inherent sampling variability also compromises microbial detection, even though blood cultures are collected under strictly sterile conditions (12). Unfortunately, in this case, the patient presented with hemorrhagic shock accompanied by infection at the time of admission. Given the emergent nature of the operative setting, obtaining additional tissue samples would have prolonged the procedure and potentially delayed life-saving interventions. Therefore, tissue sampling could not be performed. In addition, empirical antibiotics were administered before surgery because of septic shock, which probably would reduce the sensitivity of pus cultures and yield suboptimal microbiological results. In summary, we recognize that the absence of histological specimens may have limited the depth of etiological analysis to some extent, representing a limitation of our study.

A definite guide to management is still missing in the literature, and its optimal treatment remains a subject of ongoing academic debate. In confined ruptures, percutaneous catheter drainage (PCD) combined with antibiotic therapy is typically the initial treatment course. Generally, open surgery is considered only in cases of failed PCD or in non-responding patients (7). Nonetheless, abundant studies have clearly identified that the peritoneal cavity is the most common location of ruptured LA, a serious surgical emergency (24). Although surgical drainage is involved in a ruptured LA may increase morbidity, it significantly reduces mortality compared with PCD and therefore offers an irreplaceable, life-saving benefit (7). Considering the patient’s condition at admission, we believed that PCD alone would be insufficient to control the infection effectively. Furthermore, contrast-enhanced CT demonstrated peripheral enhancement of the hepatic abscess with internal septation-like enhancement. Given the patient’s critical condition and the limited amount of contrast administered, it was difficult to determine radiologically whether active bleeding was present. Therefore, any form of percutaneous drainage could have triggered or exacerbated hemorrhage and potentially posed a life-threatening risk. Therefore, emergent surgery was performed promptly while simultaneous resuscitation and antimicrobial therapy were initiated. Based on intraoperative findings and imaging, it was presumed that the moment of abscess rupture involved branches of the right hepatic artery, resulting in high-pressure arterial bleeding that rapidly led to the massive subcapsular hepatic hematoma.

In summary, for patients with PLA who present with septic shock, diffuse peritonitis, and marked elevations of total bilirubin, aspartate aminotransferase, and blood glucose, immediate concern for abscess rupture, surgery becomes the cornerstone of life-saving therapy. In those without significant comorbidities and with relatively stable hemodynamics, a laparoscopic approach can be considered as an alternative to laparotomy, achieving source control and adequate drainage while minimizing surgical trauma. Moreover, every drainage strategy should be coupled with appropriate antibiotic therapy, which remains a critical component of treatment. With fluoroquinolone-resistance rates among *Escherichia coli* (*E. coli*), *Klebsiella pneumoniae*, and other Enterobacteriaceae reaching 30% (22), the findings of Ruiz-Hernández et al. (25) and Zhang et al. (26) indicate that *E. coli* remains highly susceptible to carbapenems, displaying low resistance rates. Moreover, compared to other antibiotics, the use of carbapenems, particularly imipenem, is independently associated with lower mortality (27). Hence, in critically ill patients with severe infections, carbapenems can be initiated as empirical therapy before the pathogen is identified, with definitive adjustments made once the antimicrobial susceptibility profile of the isolate is available.

## 4 Conclusion

Rupture of the PLA is a rare but lethal complication that can rapidly evolve into septic shock and multiple organ dysfunction (6). Here we report an exceptional case of a previously healthy young woman who developed sudden hemorrhagic and septic shock due to a ruptured pyogenic liver abscess complicated by a massive subcapsular hematoma. Emergency laparoscopic débridement and drainage, combined with targeted antibiotic therapy, resulted in an excellent outcome with complete radiological resolution of the lesion. This case presents a reproducible, minimally invasive treatment paradigm for ruptured PLA, highlighting the critical importance of early recognition and prompt intervention in the absence of established guidelines. The main limitations are the single-case design without controls and the fact that rupture mechanisms and long-term outcomes still require validation in larger cohorts.

## Data Availability

The original contributions presented in the study are included in the article/supplementary material, further inquiries can be directed to the corresponding author.
